# Three-Dimensional Organoid System Transplantation Technologies in Future Treatment of Central Nervous System Diseases

**DOI:** 10.1155/2017/5682354

**Published:** 2017-08-20

**Authors:** NaiLi Wei, ZiFang Quan, Hailiang Tang, JianHong Zhu

**Affiliations:** ^1^Department of Neurosurgery, Fudan University Huashan Hospital, National Key Laboratory of Medical Neurobiology, The Institutes of Brain Science and The Collaborative Innovation Center for Brain Science, Shanghai Medical College, Fudan University, Shanghai 200040, China; ^2^Department of Neurosurgery, The Second Hospital of Lanzhou University, Lanzhou, Gansu 730030, China; ^3^Department of Neurology, The First Affiliated Hospital, University of South China, Hengyang, Hunan 421001, China

## Abstract

In recent years, scientists have made great achievements in understanding the development of human brain and elucidating critical elements of stepwise spatiotemporal control strategies in neural stem cell specification lineage, which facilitates successful induction of neural organoid in vitro including the cerebral cortex, cerebellar, neural tube, hippocampus cortex, pituitary, and optic cup. Besides, emerging researches on neural organogenesis promote the application of 3D organoid system transplantation in treating central nervous system (CNS) diseases. Present review will categorize current researches on organogenesis into three approaches: (a) stepwise, direct organization of region-specific or population-enriched neural organoid; (b) assemble and direct distinct organ-specific progenitor cells or stem cells to form specific morphogenesis organoid; and (c) assemble embryoid bodies for induction of multilayer organoid. However, the majority of these researches focus on elucidating cellular and molecular mechanisms involving in brain organogenesis or disease development and only a few of them conducted for treating diseases. In this work, we will compare three approaches and also analyze their possible indications for diseases in future treatment on the basis of their distinct characteristics.

## 1. Introduction

Stem cell therapy provides with an alternative and the last resort for curing many diseases in an extensive CNS spectrum of disease. However, poor clinical efficiency casts a showdown for stem cell therapy. In general, if neural stem cells take action, they should undergo three steps: proliferate and differentiate into due neural cells, migrate and distribute to accurate location, and integrate into host tissue and form synapse connection [1]. Unfortunately, this process usually takes several weeks [2]. Only a fringe of them finally survives and takes action. Currently, researchers and scientists devote themselves to improving the efficiency through optimizing various parameters such as engineering ideal matrices, suitable delivery approaches, and improving differentiation efficacy. However, stem cells are poorly manipulated in vivo. Once they are engrafted in vivo, they lose our control. Therefore, high-survival rate and stable environment in vivo are critical for stem cell transplantation. In order to solve these problems, organoid-like tissue might provide us with a promising approach.

Organoid is defined as a multicellular formation that spontaneously develops and self-organizes from stem cells or organ progenitors, resembling the structure and function of an organ in vivo [3]. Organoid system recapitulates the process of organogenesis in vivo and harbors stable hemostasis and architecture. Different from traditional stem cell therapy which always concentrated on specific populations of stem cells or progenitor cells, organoids provide with a complete set of cell types of an organ [3–6]. This novel therapy renders an obvious advantage over traditional stem cell therapy. Besides, this method focuses on full-functional organ-like tissue transplantation rather than purified neural cell type treatments. After engrafting into the host, they still have a stable environment in situ and can support themselves for self-renew and self-organize to integrate with host tissue. Thus, stem cells in the organoids have a higher survival rate and form functional connections with the surrounding tissue in the host [4, 7–9].

**Figure 1:**
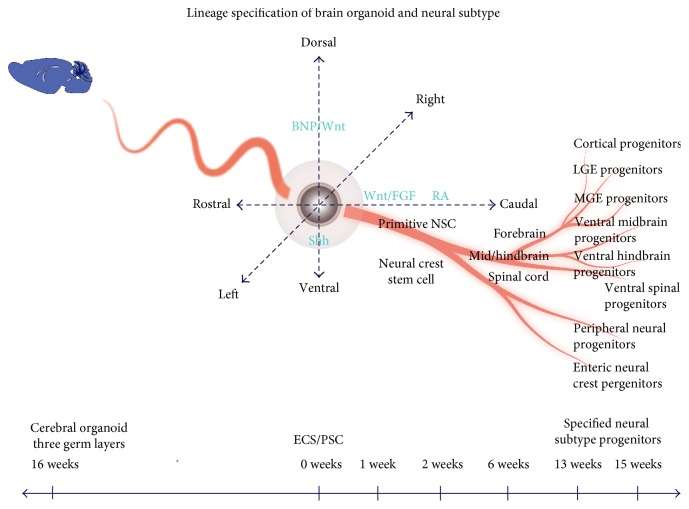
Steered with the specific spatiotemporal control strategies, a single embryonic stem cell (ESC) or pluripotent stem cell (PSC) can develop to a three germ layer brain and pure neural type. The outline has been depicted in Tao's paper (Tao et al.). BNP: bone morphogenetic protein; ESC: embryonic stem cell; FGF: fibroblast growth factor; LGE: lateral ganglionic eminence; MGE: medial ganglionic eminence; NSC: neural stem cell; PSC: pluripotent stem cell; RA: retinoic acid; Shh: sonic hedgehog.

In recent decades, neural organoid has entered into and captured our eyes. Lancaster and his team successfully established a protocol for culturing pluripotent stem cell- (PSC-) derived “cerebral organoids” that recapitulated the developing human brain's cellular organization segregates into distinct brain regions [10]. Although cerebral organoids could not fully model the organization of the brain, the method still shed a light for future treatment of diseases through organoid system transplantation which can be established in vitro culturing. In addition to Lancaster's team, several other teams developed region-specific neural organoid such as the neocortex [11], telencephalon [12], cerebellar [13], neural tube [14], pituitary gland [15], hippocampus cortex [16], optic cup [17], neural retina [18], and inner ear sensory epithelial tissue. Single embryonic stem cells (ESCs) or PSCs can be self-organized to form three-layer cerebral organoid but can also be directed to develop a region-specific neural organoid. Furthermore, they can also be manipulated and assembled to form specific morphogenesis organ (Figure 1). In specific spatiotemporal control conditions, scientists have directed ES or PSC to differentiate into both neuronal subtypes and glial subtypes. Neuronal progenitors can be specified into GABAergic, glutamatergic neurons, dopaminergic neurons, interneurons, and motoneurons [19–26], while glial progenitors can be specified into astrocytes, oligodentrocytes, and other glial subtypes [19, 27–29]. It is worthwhile mentioning that special signals can also be utilized to enhance the acquisition of the transmitter phenotype [19, 23]. These findings stretch a promising panorama for clinical treatment by distinct organoid system transplantation. Over the past decades, scientists have devoted themselves to elucidating critical element brain development and spatiotemporal control of the processes, which are extensively and fully reviewed in several perfect papers [5, 19–21, 27, 30–35]. These findings provide us with rationale and logistical feasibility to steer organogenesis to specific region. In addition, we can also design and assemble organoid to form specific morphology or function through manipulating numbers of specific stem cell types, neural network composition, numbers of receptors, and ligands. These organoids could be applied to treat central nervous system (CNS) diseases.

Although the classification of organoid was reviewed in previous papers [3], the authors focused on the purpose of organoid researches rather than the approaches to organoid formatting. Based on methods and application orientations, present review categorizes the organogenesis into three approaches. According to distinct procedure in the induction of organoid organization, we categorize these researches into three approaches (Table 1): (a) stepwise, direct organization of region-specific or population-enriched organoids [12, 18, 36–47]; (b) assemble and coculture distinct organ-specific progenitor cells or stem cells to form specific morphogenesis organoid [7, 8, 13, 17, 26, 48]; and (c) assemble embryoid bodies for induction of full layer organoid [14, 49, 50]. Besides, it will also provide with details of the examples and discuss on the rationale and logistical feasibility. In the following parts, we also compare distinct approaches and analyze for their possible indications for diseases.

## 2. Neural Organogenesis Approach

### 2.1. Stepwise, Direct Organization of Region-Specific or Population-Enriched Organoids

Currently, most researches on neural organogenesis adopt stepwise spatiotemporal control strategies to acquire neural organoids from ESCs or IPSCs [12, 18, 36–47]. In the initial stage, ESCs or IPSs are allowed to reaggregate in low-adhesion condition, namely, serum-free EB-like protocol (SFEBq), which ensures that they have enough time to proliferate and expand [47, 51]. In this stage, ESCs or IPSCs maintain pluripotency and EB-like masses harbor three germ layers (ectoderm, mesoderm, and endoderm). In the stage of neural induction, referring to neuroectodermal formation, EB-like masses are transferred to N2 medium to induce neural germ layers. Treating with exogenous signal inhibitor of BMP, Wnt, and nodal inhibitor, they can efficiently form neuroepithelial tissue, neural tube construct [14], or neocortex [42]. The early neural organoids usually display initial structure and morphology with apical-basal polarity and dorsal-ventral polarity. Further induction can promote region identity and acquire region-specific organoids. Human cerebral cortex is well structured with six layer neurons. Deep and superficial layers of neurons are distinct populations, which are connected with each other and have distinct projections and functional fate [33, 52]. As a result, with a good master of region-specific neural organoid induction technology, we can prepare specific population of progenitor cells which we want to perform cell replacement therapy. Although we cannot get purified cell population, high number of specific neural population can be acquired through using this method [13, 23, 39, 42]. Thus, this organogenesis approach will be neural type population orientated.

**Figure 2:**
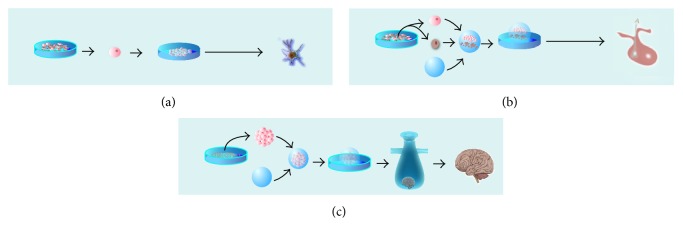
Schematic of three neural organogenesis approaches in vitro. (a) Stepwise, direct organization of region-specific or population-enriched organoids; (b) assemble and direct distinct organ-specific progenitor cells or stem cells to form specific morphogenesis organ; (c) assemble embryoid bodies for induction of multilayer organoids.

With the support of specific spatiotemporal control strategies, this approach efficiently directs ESCs or PSCs differentiate into and self-organize region-specific neural organoids with a high number of specific progenitor cells (Figure 2(a)). Both intrinsic and extrinsic signals are involved in the regulations. During embryonic days 9 and 10, corticogenesis in mice takes places in a polarized epithelium with its apical surface forming the lumen of the tube (future ventricles). Early cortical neural stem cells (NSCs) divide symmetrically. At E11, NSCs begin to divide asymmetrically. One daughter cell retains its NSC identity while the other becomes a neuron. Early-born neurons form the deep layers of the cortical plate (layers 5 and 6), and later-born neurons migrate outward past the deep layers to establish the superficial or upper layers (layers 2–4). Although this neural induction process seems to be a cell fate program, it could be manipulated by this approach. For example, Muguruma et al. [13] reported that they acquired polarized cerebellar plate in 3D culture with a stepwise spatiotemporal control strategy. Firstly, they dissociated ESCs at day 0. In order to promote neuroectodermal differentiation, they inhibit mesenchymal differentiation by addition of the transforming growth factor β- (TGF-*β*-) receptor blocker. On days 2–14, ESCs were treated with FGF2 and insulin with the aim to be steered to differentiate into cerebellar progenitors. On day 14, additional FGF19 and SDF1 treatments induced progenitors to self-form cerebellar plate neuroepithelial structures with dorsal-ventral polarity. After these treatments, neuroepithelial rosettes had transformed into large and continuous flat-oval structures with the apical side inward regarding the ova. Admittedly, major portion of the cerebellar plate neuroepithelium generates Purkinje cells and interneurons and they finally acquired those electrophysiologically functional Purkinje cells.

Muguruma et al.'s success displays a good example which shows how scientists manipulate lineage of organogenesis. However, it will be the tip of an iceberg in the future. Recently, considerable excellent review papers have mapped neural subtype specification lineage and fundamental developmental principles [19–21, 28, 30, 32, 53]; based on which, we can briefly conclude as following: (1) Early cortical neural stem cells (NSCs) residing in a polarized epithelium divide symmetrically at their early expansion. At E11 in mice, NSCs begin to divide asymmetrically, generating one neuronal progenitors and the other continuing maintaining NSC identity. In this stage, apical surface forming the lumen of the tube (future ventricles), early neural progenitors migrate up and down within the ventricular zone (VZ) of the neuroepithelium. Neuronal daughters detach and migrate to subventricular zone (SVZ). (2) Although neocortical excitatory or inhibitory neurons can be generated in both VZ and SVZ, different regulator factors still determine their subtypes. Cux2 and Cux2+ excitatory progenitors, respectively, generate distinct subtypes of upper-layer and deep-layer neurons. SST+ or PV+ progenitors result in inhibitory neurons in all layers except layer Ι whereas CR+ or VIP+ cells give rise to inhibitory neurons particularly abundant in layers IV, III, and II. NPY+-derived cells could be found in all cortical layers; transcription factor FOXA2 is critical for midbrain DA neuron development while coexpressions of the floor plate (FP) marker FOXA2 and the roof plate marker LMX1A are as well required. (3) Astrocytes in the cerebral cortex are produced from the cortical ventricular zone (VZ) or from the ventral forebrain. In addition, glia of the cerebral cortex is also produced from the postnatal SVZ, a specialized reservoir of glial and neuronal progenitors. Almost all of the neural subtype specification can be mapped in recent year researches [19] and could be manipulated to induce what we want (Figure 1).

Neural organogenesis via this approach is always neural population orientated. Also, it is expected to efficiently acquire specific neural progenitors or stem cells after region-specific differentiation induction. The region-specific identity, regulated by cell surface signals, is important for neural network reconstruction [54] in the process of neuronal self-recognition and non-self-discrimination. Differentiation is directed by series of distinct epigenetic mechanisms [55–57]. In the stage of stepwise induction, ESCs or IPSs gradually lose their differentiation pluripotency due to DNA methylation and losing GC [56]. Distinct region specificity may have distinct cell identity. Although these specific neural stem cells are not purified, they still maintain their region identity ability [55]. The neurons with the same specific region identity easily connected with each other [58]. After they are engrafted to the specific region in vivo, they might easily establish neural network with local neurons. By contrast, neurons without an additional common factor have to take time to reconstitute their neural network. Compared with purified stem cell population transplantation, this population-enriched organoids may have a high-survival rate and efficiency to form mature synapse connections.

However, the organoids lost the potentiality to form multiple germ layer structure because stepwise differentiation induction steers the series of transcriptional regulators and DNA methylation to specific germ layer structure. Although stepwise strategy can induce initial neural cystic formation, this method might not construct sophisticated morphology efficiently [48]. Majority of these organoids can only form simple structures.

### 2.2. Assemble and Direct Distinct Organ-Specific Progenitor Cells or Stem Cells to Form Specific Morphogenesis Organ

This approach has been extensively applied to generate organoids with complex morphology, such as the pituitary [7, 8], optic cup [17], feather buds [59], salivary gland [60], hair follicle [61, 62], gingival tissues [63], and tooth [64]. These tissues usually locate in the transition region among distinct structure layers, and their organoid formation requires communication among the distinct region tissues. However, one induction condition only steers specific region identity. In order to acquire these organoids with complex architecture, they assemble two or more distinct populations of progenitors or stem cells in the 3D matrix and coculture under a specific differentiation induction condition [7, 8, 13, 17, 26, 48]. This approach is defined as a term of “self-assembly” by Sasai [65]. Briefly, it refers to the spontaneous formation of a patterned organ with multistructure and multicellular by selective aggregation of cells or by rearrangement of the relative positions of cells within the structure [65]. Through assembling two or more cell types in a 3D culture, this method is sought to recapitulate an interactive microenvironment and mimic multicellular or multistructure level in the vivo organogenesis. For example, pituitary gland consists of neurohypophysis and adenohypophysis (Figure 2(b)). Adenohypophysis (anterior and intermediate lobes of the pituitary gland) contains several types of endocrine cells while neurohypophysis (posterior pituitary) consists of the axons and secretory termini of hypothalamic vasopressin and oxytocin neurons. In order to resemble structure of the pituitary gland, Suga et al. [8] detached outer component of epithelium cells of day 6 aggregates and cocultured them with inner neuroepithelial cells treating with hedgehog signaling. They found that this synthetic approach could successfully generate pituitary endocrine cells. At the interface of these two epithelia, Rathke's-pouch-like three-dimensional structures generated earlier. Functional organ bud was constructed in vitro, which was proved by the evidence that various endocrine cells efficiently secreted hormone in response to corticotrophin-releasing hormone after grafted in vivo. Based on the same approach, Eiraku's team achieved another success by reconstructing functional optic cup in vitro [17] and mimicking the multistructures of the optic cup consisting of the outer (pigmented) and inner (neurosensory) layers of the retina.

In addition to the pituitary gland and optic cup, the cortex in the central nervous system (CNS) also illustrates a prime example of an organ with extreme neuronal diversity and multilayer structures. Cell types of the cortex are broadly classified into excitatory projection neurons (PNs) and inhibitory interneurons (INs). This approaches might be applied to assemble PNs and INs in ratio, mimicking the vivo structures so as to allow enhanced cortical plasticity in the corticogenesis. Moreover, the process of spontaneous formation of ordered patterns and structures from a population of elements promotes functional connection with each other. Through bridging connections among neurons, glial cells and the vasculature, astrocytes provide with microenvironment and homeostatic processes for neuronal regeneration. Coculture astrocyte progenitors and neural stem cell might promote neurogenesis and synaptic connections [66–68]. Pouchelon et al. [69] found that functional differentiation of postsynaptic L4 neurons and cognate intracortical circuits were associated with TC-input-type-specific control. In addition, the finding also instructs the development of modality-specific neuronal and circuit properties during corticogenesis and shows another example of interactive communications among cellular levels. Due to these evidences, assembling multiprogenitors or differentiated PSCs or ESCs facilitates neurogenesis and functional connections. The approach appears more suitable for the organogenesis, requiring multicellular or multistructure interactive communications.

Assembling neural subtypes in the neural organoids also plays an important role because also it is important to reprogram the subtype diversity so as to promote the generation of functional neural circuit in the self-organization tissues. Distinct projecting neurons choose highly selective synaptic connectivity, both pre- and postsynaptic, within the same local circuits [70]. Both postsynaptic target of inhibitory interneurons and the properties of their synaptic connections depend on the identity of their projection partners [70]. Emerging data demonstrate that projection neurons and interneurons might “chemical match” for the development of excitatory and inhibitory cell assemblies [20]. Meanwhile, synaptic input also has the capability to affect specific neuron subtype differentiation during cortical circuit assembly [69]. Astrocytes comprise up to 40% of all CNS cells, which not only provide support to neurons but also actively regulate synapse formation and maturation [71]. Consequently, it appears a critical role of the assembling way of specific neural subtypes in establishing the neural circuit in the organoid.

### 2.3. Assemble Embryoid Bodies for Induction of Multilayer Organoids

Figure 2(c) illustrates procedures of this approach. After 4-day suspension culture, ESCs or PSCs aggregate and form embryoid/embryoid-like bodies. The procedure in vitro culture recapitulates the key events of embryogenesis in vivo to obtain the three developmental germ layers from which all cell types arise [4, 17, 50, 72]. The cell pellets are entrapped in a droplet of matrigel or collagen to coculture for and differentiate to develop a specific organ in a specifying differentiation strategy. Through manipulating extrinsic signal modulation, scientists can germ layer specification and cell differentiation [73]. In addition, embryoid bodies with three germ layers could also differentiate into functional tissue-specific cells with three germ layers. Takashi Tsuji's team designed a clustering-dependent embryoid body transplantation plan to develop a 3D integumentary organ system. In the system, formation involves three germ layers of cell types, respectively, dermis, hair follicles and sebaceous glands. After transplantation, hair follicles successfully generated with fine connections with the surrounding tissues such as the epidermis, arrector pili muscles, and nerve fibers, without tumorigenesis. Takagi et al.'s work provides not only a good example for assembling embryoid body approach but also an example for future application orientation that it appears to be suitable for the organogenesis involving more than one germ layer.

Different from other organoid induction approaches, this approach resorts to acquire specific organoid with full layer structure. Researchers adopt this approach to investigate natural organ development procedures or mechanisms involving diseases [4, 17, 50, 72]. Unlike EB-like aggregation in SFEBq procedure, this approach prolongs the culturing time of EB-like population. Additionally, they assemble and coculture EB-like populations in 3D matrix in order to induce self-organization and morphogenesis.

At present, only a few neurological scientists focus on neural organogenesis or cerebral organoid with multiple germ layers [50] because the overwhelming majority of them hold the theory that neural organ induction starts in neuroectodermal stage. Lancaster et al. developed a cerebral organoid in vitro based on this method. Cerebral organoids showed recapitulate features of human cortical development, namely, characteristic progenitor zone organization with abundant outer radial glial stem cells. Most brain tissues derived from neuroectodermal layer whereas mesoderm and endoderm germ layers involve neural organogenesis. Formation of three germ layers cannot be isolated from each other. The germ layers are defined by their position at the stage of late gastrula. At the late stage of embryogenesis, their regional divisions are no longer distinct [74]. Cardiovascular and cerebrovascular derived from mesoderm germ layer stretch throughout the body including the brain and transport blood and energy. Nervous system originated from neuroectoderm forms parasympathetic and sympathetic nervous systems and governs the function of the cardiovascular system [75]. Therefore, they are supported by each other and connected with each other. Mesenchymal stem cells derived from mesoderm germ layer could also be applied in the degenerative neurological diseases [76–78]. It was found that human mesenchymal stem cells (hMSCs) in culture could provide humoral signals that selectively promote the genesis of neurons and oligodendrocytes from NSCs [68]. In addition, MSC could differentiate into neuron-like cells as well as by a competence to generate a “neuroprotective” environment [79]. This approach may facilitate local reconstitution of vascular networks. Considering the above discussion, we can make a speculation that the assembling embryoid body approach might be applied to generate a cerebral organoid with multiple germ layers. The organoid is more probably suitable for treating patients with multilayer brain tissue loss including traumatic brain injury, stroke [80], hemispherectomy, or lobotomy because of tumor, epilepsy, and intracranial hematoma.

## 3. Similarities and Differences

### 3.1. Similarities

Neural organogenesis is regulated by a series of epigenetic regulators. In order to develop to an organoid, a single cell in all approaches has to undergo spatiotemporal steering process. Neurons differentiate and migrate to specific regions and layers along anterior-posterior (AP) and dorsal-ventral (DV) axis [5] and are regulated by various regulator factors. Wnt, FGF, and retinoic acid (RA) are responsible for their caudalizing activity in the embryological context; Shh signaling for ventralization of embryonic neural tissue; BMP and Wnt signaling for dorsalization [5]. Besides, time order also determines the locations of neurons. Pioneer neurons are the earliest-born neurons in the cortex and then followed by deep cortical layers VI and V, then by upper layers IV, and lastly layers II/III [81]. Late-born neurons tend to localize more basally to early-born neurons [11]. All the three approaches possess the common epigenetic regulating factors. Even though neural organoids in the three approaches are different from each other in composition and structure in the organoids, they have a common neuroectodermal induction process.

In addition, all approaches adopt 3D culture to mimic in vivo microenvironment to provide a scaffold and niche for stem cells to aggregate, attach, and form organoids. Biochemical and biophysical signals are also involved to steer organogenesis in all three approaches. These signals determine organogenesis microenvironments consisting of a complex array of signaling mechanisms from niche support cells, the ECM, and mechanical forces, as well as systemic and physiochemical conditions such as oxygen and pH levels [82]. For example, the identity of PSCs is associated with local oxygen concentration and hypoxia inducible factor-1*α* (HIF-*α*) plays a distinct and stage-specific roles in reprogramming human cells to PSCs [83] and involves in angiogenesis and stem cell maintenance. NSCs within the SVZ maintain the integrity of their vascular niche through HIF-1-mediated signaling mechanisms [84]. Relief of hypoxia in developing the cerebral cortex by growth of blood vessels temporospatially coincided with NSC differentiation [85]. Considerable biophysical factors such as adhesion and viscoelastic and stress relaxation of extracellular matrices take impact not only on cell spreading and proliferation but also on the differentiation to specific cell types [86–89]. Biophysical cues also generate a change in protein conformation in response to tension or compression and thus to take effect on the cell formation [87]. All of these signals could be manipulated for lineage of specific organ. Currently, a three-dimensional culture is widely applied in organogenesis. In 3D organoid culture system, it allows the formation of brain tissues through either self-assembly or active induction. Some scientists attempt to display several subtype stem cells in ratio or in multilayer in order to mimic the ratio or structures in vivo and finally acquired full functional organ [4, 61]. With the support of 3D organoid culture, scientists have the possibility and opportunity to rewrite the structure or composition of organogenesis program in vitro.

### 3.2. Differences

Neural organoids via the first approach are specific region orientated. ESCs or IPSCs can be stepwise, induced to differentiate into an organoid with high number of neural populations. These populations of neural stem cells are not purified cells. Instead, the organoids consist of several region-specific neural populations with special cell surface marker. These cells can form specific morphology and structure [18, 39, 40]. Additionally, they can organize local neural connections among distinct populations [13, 40]. By treating with specific markers, neural organoids could be dissociated to collect purified stem cells or progenitor cells. Therefore, we can efficiently acquire purified neural cell populations with region identity via the first approach induction.

Via the second approach, neural organoids are specific morphology orientated. The organoids usually consist of several anatomic parts. Anatomically, the morphogenetic self-organization locates in the cross-connection area among distinct regions and requires coculture of distinct populations of neural cell populations. Coculture can steer these parts to generate functional and morphologic connections. In order to promote the morphogenesis, distinct populations with different region identity were assembled in 3D droplets. As a result, the ratios among distinct populations, the matrix composition, biophysical, and biomechanical parameters need to be designed precisely mimicking in vivo process. This approach has made higher requirements for assembling protocol. However, this neural organoids have specific indications for diseases. The organoids could be engrafted into the brain as an integral preorgan. However, size and morphology of these artificial organoids have to match with host tissue. Otherwise, they are being potential occupying lesion.

Neural organoids via the third approach are full-germ layer orientated. They have more complex and full structure and morphology. At present, researches focus on the development of brain structure [14, 49, 50] and few of them have successfully mimicked the brain structure generation in vivo although Li et al. reported a folded cerebral organoid with simple structure [49]. This approach aims to acquire not only full function but also both integral structure and morphology. Cell populations in the neural organoids involve not only neural populations but also cell populations derived from other germ layers such as vessels and the immune system. However, how to reconstruct the cerebrovascular and immune system in the organoids still remains to be solved.

## 4. Clinical Treatment Consideration

### 4.1. Region-Specific or Population-Enriched Organoids

Stem cell therapy is a promising approach to replace damaged cells in the brain or replenish losing cells in the nucleus [90–92]. In a variety of neuronal degenerative diseases, patients have specific neural population loss or damaged. In Parkinson's disease (PD), midbrain dopamine (DA) neurons, especially innervating motor neurons, are degenerated at least at an early stage. However, Huntington's patients gradually lose their medium spiny GABA (*γ*-aminobutyric acid) neurons in the striatum. Motor neuron loss could also be observed in spinal muscular atrophy (SMA) and amyotrophic lateral sclerosis (ALS) patients [19]. The specific neural subtypes are preferentially affected and degenerated, so few pharmacy drugs could curb the pathological insult progress. Neural stem cell therapy improves these diseases not only in animal models but also in clinical trials [77, 93–98]. Traditional purified cell therapy has low clinical efficiency. However, these neural populations generated by the first neural organoid induction approach could improve its treatment efficiency. These populations in the neural organoids have specific region identity. After engrafting in to the host brain, they could connect with local neurons in an efficient way. Thus, neural organoids via the first approaches are more suitable for these diseases with specific neural type loss or damage.

### 4.2. Assembled Specific Morphogenesis Organoids

These neural organoids are specific morphology orientated and theoretically suitable for treating these diseases with specific neural structure damaged or atrophy. For example, Sheehan's syndrome always follows after pituitary atrophy which results from postpartum bleeding and pituitary tumor or surgery [99, 100]. Traditional treatment with pharmaceutical drug has several adverse effects. These patients might have another alternative treatment by transplantation of artificial pituitary induced by assembling and coculturing hypothalamic as well as oral ectoderm stem cells [7]. Other similar diseases can be optic atrophy [17], retinal diseases [18], and so on. In addition, peripheral nerves are other potential indications for the second neural organoids. Schwann cell in the peripheral nervous system is derived from the neural crest. Maturity of Schwann cell requires interaction among the Schwann cell and peripheral tissues [101]. Skin-derived precursor cells facilitate the regeneration process of peripheral nerve [102]. In addition, coculture of progenitor cells of peripheral tissues and neural stem cells might promote the generation of peripheral nerves.

### 4.3. Assembled Multilayer Organoids

Actually, these neural organoids are a preorgan with integral structure and function and can treat these diseases with structure loss or damage. These patients might have an integral structure loss of brain region because of traumatic brain injury, stroke, hemispherectomy, or lobotomy caused by tumor, epilepsy, and intracranial hematoma. There are no niches for stem cells to attach. Therefore, organoids have to support by themselves. Before engrafted to the host brain, the neural organoids must generate a preorgan with full structure.

## 5. Conclusion

To conclude, 3D organoid system transplantation renders obvious advantage over traditional approaches which probably focus on pure populations of particular stem cell-derived cell types. Instead, 3D organoid system resembles natural self-formation process of specific organ through assembling cell subtypes, layers, cell subtype proportion, and manipulating biophysical signals. These strategies promote correct connections among multilayer and multicellular synapses and establishment of local neural circuits. In comparison with conventional therapy, 3D organoid system transplantation promotes stem cell survival and functional connection after grafting in vivo [4, 8, 17, 61, 64]. Although 3D organoid system transplantation was reported to treat CNS diseases only in a few papers [4, 8, 17, 61, 63, 64], it still appears to be promising in the future treatment. There are three approaches in neural organoid which could be applied, choices of which can be determined depending upon due diseases. The first organogenesis approach is the region-specific or population-enriched organoids which refer to the fundamental method. We could acquire specific neural subtypes or specific organ, which could be applied to treat neuronal degenerative diseases, such as Parkinson's disease, Huntington disease, ALS, and SMA. The second synthetic approach designed in the multicellular level or multiculture level can generate functional self-formation tissue to treat neural organ-associating functional disorders such as pituitary gland atrophy and optic cup loss. Peripheral nerve damage could also be treated by this organoids. The organogenesis approach by assembling embryoid bodies for specific organ is theoretically more suitable for patients with total layer tissue loss, such as traumatic brain injury, stroke [80, 103], hemispherectomy, and lobotomy because of tumor, epilepsy, and intracranial hematoma. In order to promote local functional connections, scientists should design the neural subtype diversity in the process of in vitro organoid induction, matching the ratio between excitatory and inhibitory neurons, neurons and astrocyte, and input synapses and output synapses.

## Figures and Tables

**Table 1:** Related research.

Author	Published year	Organoid induction designs	Organoid type	Induction condition factors	Culture medium	SFEBq procedure
*Approach 1: Stepwise, direct organization of region-specific or population-enriched organoids*						
Lakshmi Subramanian	2017	Neural tissue samples between GW8 and GW10; coronal vibratome sections were transferred to culture medium.	Forebrain	Wnt inhibitor; TGF-*β* inhibitor; high O_2_ penetration; concentration of matrigel	Cortical slice culture medium	No
Marina Bershteyn	2017	hIPSCs were cultured in using cortical differentiation medium.	Cerebral organoids	Rho kinase inhibitor; Wnt inhibitor; TGF-*β* inhibitor	Cortical differentiation medium; N2 culture medium	No
Adrian Ranga	2016	ESCs were cultured in high-throughput combinatorial screening of 3D microenvironments; stepwise induced.	Cyst-like structure neural tube with apical-basal polarity	RA; sonic hedgehog; synthetic nondegradable materials	Neurobasal medium	No
Lixiong Gao	2016	hESCs were cultured with SFEBq procedure; H1 cells were selected to induce organoids.	Neural retinal tissue	Wnt signal inhibitor; SAG; CHIR99021; N2; retinoic acid; high oxygen concentrate (40%)	N2 culture medium	Yes (3 days)
Hideya Sakaguchi	2015	hESCs were cultured in SFEBq culture for 73–84 days and dissociated with neural tissue dissociation kit and then cultured in neurobasal medium.	Hippocampal tissue	Wnt inhibitor; TGF-*β* inhibitor; N2 supplement; chemically defined lipid; neurobasal medium; bone morphogenetic protein; Wnt	N2; neurobasal medium	Yes (3 days)
Anca M. Paşca	2015	hCSs were dissociated to culture to induce organoids.	Cortical tissue with functional neural network	BMP inhibitor and TGF-*β* inhibitor; NT3; BDNF; FGF2; EGF; neurobasal; dorsomorphin; SB-431542	Neural medium	No
Atsushi Kuwahara	2015	Elective NR differentiation from hESCs.	Neural retina	BMP4-inhibiting GSK3 and FGFR; Y-27632; gfCDM; CHIR99021; SU5402	N2; neural retina medium; retinosphere medium	No
Karl R. Koehler	2013	ESCs were dissociated to induce organoids.	Inner ear sensory epithelial tissue	BMP4; TGF-*β* inhibitor FGF2 and LDN; N2B27 medium; N2 medium; SU5402; SB-431542	N2B27 medium	Yes (3 days)
Taisuke Kadoshima	2013	Stepwise induction of neocortex with high numbers of pyramidal neurons.	Neocortex	Rho kinase inhibitor, TGF-*β* inhibitor, and Wnt inhibitor; B27, N2, and chemically defined lipid concentration; hedgehog signals	N2 and chemically defined lipid concentration	Yes (3 days)
Lucy A. Crompton	2013	Neurospheres were dissociated cholinergic neurons to culture in NEM.	Forebrain cholinergic neurons	Nodal/TGF-*β* signaling inhibitor, ROCK inhibitor Y-27632, FGF2, and EGF	Modified chemically defined media, NEM	Yes (4 days)
Yichen Shi	2012	Neuroepithelial cells were dissociated to induce organoids.	Cerebral cortex with projection neurons and neural networks	SB431542, FGF2, noggin, neurobasal, Y-27632	N2B27 (N3) medium; N2	No
Jessica Mariani	2012	Undifferentiated PGP1-1 and colonies were dissociated into single cells to induce organoids.	Early forebrain	N2 supplement, Y-27632, FGF2, Wnt inhibitor DKK1, BMP inhibitor BMPRIA-Fc, TGF-*β*/activin/nodal inhibitor SB431542	N2	Yes (3 days)
Teruko Danjo	2011	Foxg1: venus^+^ cells were sorted to stepwise, induce to ventral telencephalic tissues.	Ventral telencephalic tissues	BDNF, vibratome, Shh	Neuron culture medium; NT3; neurobasal/B27	Yes (3 days)
Mototsugu Eiraku	2008	ESCs were sorted to induce of polarized cortical neuroepithelia.	Cortical tissues	FGF, Wnt, and BMP; N2 medium, KSR	Cortical slice culture medium	No
Kiichi Watanabe	2005	Stepwise induction of neocortex with high number of telencephalic precursors.	Cortical organoids with high number of telencephalic precursors	Dkk1, Bf1. LeftyA, Wnt3at, Wnt, and nodal	Cortical slice culture medium	Yes (3 days)
*Approach 2: Assemble and direct disctinct organ-specific progenitor cells or stem cells to form specific morphogenesis organ*						
Chikafumi Ozone	2016	Coculture specific ventral hypothalamic NE tissue and nonneural ectoderm formation.	Anterior pituitary	KSR; FGF2; BMP4	gfCDM	Yes (3 days)
Keiko Muguruma	2015	hESCs were cultured to form neural tube-like NE structures and dissociated with anti-KIRREL2 antibody and then cocultured with RL tissues to generate Purkinje cells.	Polarized cerebellar tissue and Purkinje cells	SDF1 and FGF19; FGF2	gfCDM; N2	Yes (3 days)
Andrea Meinhardt	2014	Assembled R1 mESCs, IB10 mESCs, and 46C in 3D matrigel matrix and cocultured to induce the formation of neuroepithelial cyst.	Neuroepithelial cyst	RA, SAG, cyclopamine	N2B27 medium	No
Hidetaka Suga	2011	Assembled nonneural ectoderm and hypothalamic neuroectoderm cells in three-dimensional culture and cocultured to induce of adenohypophysis like tissue.	Adenohypophysis	SAG, DAPT, BIO, Wnt4, and Wnt5, FGF8, Nodal, IWP2, FGF10	CDM medium; DAPT-free medium	Yes (10 days)
Mototsugu Eiraku	2011	Assembled neuroectodermal epithelium tissue and nonretinal neuroectodermal epithelium in three-dimensional culture and cocultured to induce of optic cup.	Optic cup	40%-O2/5%-CO2, N2, RA, CUY21 generator	N2	Yes (3 days)
Xue-Jun Li	2008	Cultured neuroepithelial cells to generate motoneurons and then coculture of motoneurons and myocytes.	Ventral spinal tissue	RA and Shh, BDNF, GDNF, IGF1, Shh, B27	Neural differentiation medium; N2B27	No
*Approach 2: 2.3 Assemble embryoid bodies for induction of multilayer organoids*						
Yun Li	2017	Differentiation of hESCs to EBs. EBs were embedded in droplets of matrigel. Embedded EBs were subsequently cultured to induce cerebral organoids.	Cerebral organoids	bFGF; ROCK inhibitor; orbital shaker; WIBR3; BDNF	Neurobasal; N2	Yes (6 days)
Madeline A. Lancaster	2013	Assembled EBs in the droplets of matrigel and cocultured to induce the formation of cerebral organoids.	Cerebral organoids	Retinoic acid, protein kinase (ROCK) inhibitor, neurobasal, N2, insulin, B27	N2B27	Yes (6 days)
